# Thermodynamic Cards of Classic NADH Models and Their Related Photoexcited States Releasing Hydrides in Nine Elementary Steps and Their Applications

**DOI:** 10.3390/molecules30051053

**Published:** 2025-02-25

**Authors:** Bao-Chen Qian, Xiao-Qing Zhu, Guang-Bin Shen

**Affiliations:** 1 College of Medical Engineering, Jining Medical University, Jining 272000, China; qianbc@mail.jnmc.edu.cn; 2 Department of Chemistry, Nankai University, Tianjin 300071, China

**Keywords:** thermodynamic cards, photoexcited NADH models, organic hydrides, elementary step thermodynamics

## Abstract

Thermodynamic cards of three classic NADH models (XH), namely 1-benzyl-1,4-dihydronicotinamide (BNAH), Hantzsch ester (HEH), and 10-methyl-9,10-dihydroacridine (AcrH), as well as their photoexcited states (XH*: BNAH*, HEH*, AcrH*) releasing hydrides in nine elementary steps in acetonitrile are established. According to these thermodynamic cards, the thermodynamic reducing abilities of XH* are remarkably enhanced upon photoexcitation, rendering them thermodynamically highly potent electron, hydrogen atom, and hydride donors. The application of these thermodynamic cards to imine reduction is demonstrated in detail, revealing that photoexcitation enables XH* to act as better hydride donors, transforming the hydride transfer process from thermodynamically unfeasible to feasible. Most intriguingly, AcrH* is identified as the most thermodynamically favorable electron, hydride, and hydrogen atom donor among the three classic NADH models and their photoexcited states. The exceptional thermodynamic properties of XH* in hydride release inspire further investigation into the excited wavelengths, excited potentials, and excited state stabilities of more organic hydrides, as well as the discovery of novel and highly effective photoexcited organic hydride reductants.

## 1. Introduction

**Scheme 1:**
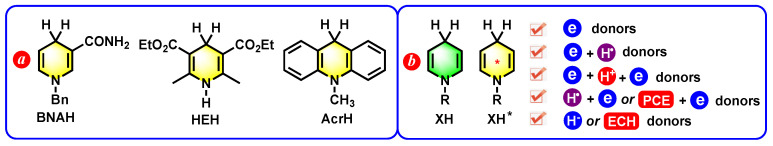
(**a**) The chemical structures of BNAH, HEH, and AcrH; (**b**) XH and XH* act as electron, hydrogen atom, and hydride donors in chemical reactions (PCE refers to proton-coupled electrons and ECH refers to electron-coupled hydrogen atoms).

Nicotinamide coenzyme is a vital oxidoreductase in vivo, participating in numerous essential enzymatic reactions as a hydride carrier [1,2]. Many organic hydrides (XH) were designed and synthesized as NADH models to mimic the outstanding reducing properties of NADH coenzyme in vitro [3]. Among them, 1-benzyl-1,4-dihydronicotinamide (BNAH), Hantzsch ester (HEH), and 10-methyl-9,10-dihydroacridine (AcrH) are the most classic NADH models, featuring a typical 1,4-dihydropyridine structure (Scheme 1a), whose redox properties have been extensively studied and well established in previous literature [3,4]. Moreover, photoexcited organic hydrides (XH*) have been discovered and verified to exhibit superior reducing activity, characterized by extremely lower oxidation potentials and stronger thermodynamic driving forces for releasing hydrogen atoms or hydrides [5,6,7].

**Scheme 2:**
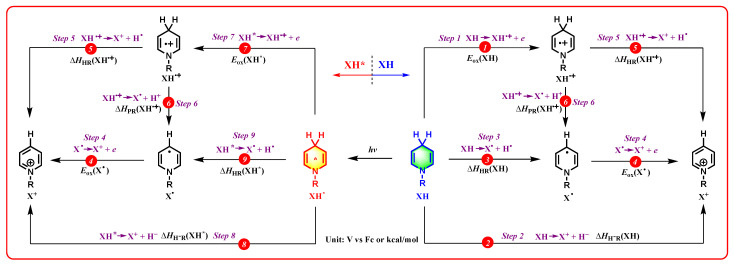
The nine possible elementary steps of XH and XH* releasing hydrides.

Until now, organic hydrides (XH) [8,9,10] and photoexcited organic hydrides (XH*) [5,6,7], particularly BNAH, AcrH, HEH, and their photoexcited states (BNAH*, AcrH*, and HEH*), are not merely used as hydride or hydrogen molecule (H_2_) reductants for reducing unsaturated compounds. As hydride carriers, organic hydrides (XH) [8,9,10], especially photoexcited organic hydrides (XH*) [5,6,7], can provide electrons, hydrogen atoms, protons, or hydrides (Scheme 1b) to generate key intermediates such as radical anions, radicals or anions. These intermediates subsequently undergo chemical transformations to construct various molecules with structural complexity. In fact, BNAH, AcrH, and HEH can be easily excited to their photoexcited states (BNAH*, AcrH*, and HEH*) in solution, and have been widely applied into photochemical synthesis [5,6,7,8,9,10,11,12,13,14,15,16,17,18,19,20,21,22,23,24,25,26,27]. Since the process of XH or XH* acting as reducing reagents in chemical reactions may involve multiple mechanisms (Scheme 1b), including e [11,12,13,14,15,16], e + H^•^ [17,18,19,20,21,22,23], e + H^+^ + e [24,25], H^•^ + e [26], H^−^ [9,10,27,28], and nine possible elementary steps (Scheme 2), the corresponding nine thermodynamic driving forces for XH or XH* releasing hydride are crucial and urgently desired thermodynamic parameters to enable chemists to predict and evaluate the characteristic properties of various reaction intermediates [9,10,11,12,13,14,15,16,17,18,19,20,21,22,23,24,25,26,27,28] and elucidate the reaction mechanisms.

Our group has been committed to determining the thermodynamic data associated with the release of hydrides from organic hydrides in acetonitrile [3]. In our previous work, the six possible thermodynamic driving forces of BNAH, HEH and AcrH releasing hydrides in acetonitrile were experimentally determined in a lab [3,4]. Given the vital applications of BNAH*, HEH* and AcrH* into photochemical synthesis as hydride carriers [5,6,7,8,9,10,11,12,13,14,15,16,17,18,19,20,21,22,23,24,25,26,27], herein, the thermodynamic cards for the three classic NADH models, i.e., BNAH, HEH and AcrH, as well as their related excited states (BNAH*, HEH* and AcrH*) releasing hydrides (Scheme 2) are established, aiming to elucidate and understand the reducing abilities of XH, XH* and related intermediates, and to further demonstrate their applications in reduction reactions.

## 2. Results and Discussion

### 2.1. The Definitions, and Sources of Nine Potential Thermodynamic Driving Forces for XH and XH* Releasing Hydrides in Acetonitrile

For XH and XH*, there are a total of nine possible elementary steps (*Steps 1–9*) involving in the release of hydrides, which are clearly illustrated in Scheme 2.

Specifically, *Step 1* and *Step 4* in Scheme 2 represent the chemical processes of XH and X^•^ releasing electrons, described by the equations XH → XH^•+^ + e and X^•^ → X^+^ + e, respectively. The thermodynamic driving forces of *Step 1* and *Step 4* are defined as the oxidation potentials of XH and X^•^, denoted as *E*_ox_(XH) and *E*_ox_(X^•^), which are determined by electrochemical experiments in a lab.

However, due to the highly unstable nature of X^•^ in solution, direct measurement of *E*_ox_(X^•^) is not feasible. Additionally, the reduction potentials of X^+^ and oxidation potentials of XH are irreversible in acetonitrile. Therefore, the potential determinations were performed using a combination of Cyclic Voltammetry (CV) and Osteryoung Square Wave Voltammetry (OSWV) methods simultaneously via an electrochemical apparatus (BAS-100B) under an inert atmosphere of Ar or N_2_ in deaerated acetonitrile to ensure accurate and reliable results [3,4].

Numerous studies have confirmed that the OSWV method is a more precise electrochemical technique for evaluating the single-electron redox potentials of substrates with irreversible electrochemical processes compared with the CV method, with uncertainties potentially as low as a few tenths of a millivolt [29,30]. Accordingly, the oxidation potential of X^•^ is equivalent to the reduction potential of X^+^, *E*_ox_(X^•^) = *E*_red_(X^+^), and the *E*_red_(X^+^) can be easily and directly determined using the OSWV method.^3–4^ In this context, the standard oxidation potential values of XH and X^•^, *E*_ox_(XH) and *E*_ox_(X^•^) in acetonitrile were derived from our previous electrochemical experiment using the OSWV method [3,4]. Utilizing the OSWV method, the single-electron redox capabilities of 421 organic hydrides and unsaturated compounds were measured and evaluated in acetonitrile [3]. Based on their redox potentials and hydricities, thermodynamic cards for these 421 organic hydrides and unsaturated compounds were established, detailing their abilities to release or accept hydrides. These thermodynamic cards have been successfully applied to reduction reactions, providing valuable insights into their reactivity and potential applications [3]. 

*Step 2* in Scheme 2 is the chemical process of XH releasing hydrides, described by the equation XH → X^+^ + H^−^. The thermodynamic driving force of *Step 2* is described as the molar enthalpy change of XH releasing hydrides, denoted as Δ*H*_H_^−^_R_(XH) (where the subscripted “H^−^R” refers to hydrides release throughout the text). Δ*H*_H_^−^_R_(XH) values were obtained by determining the molar reaction heat (Δ*H*_r_) between XH and hydride acceptor (Y^+^), whose molar enthalpy change of Y^+^ accepting hydrides Δ*H*_H_^−^_A_(Y^+^) is well established and determined using isothermal titration calorimeter (ITC) in acetonitrile. The molar reaction heat (Δ*H*_r_) is defined as the molar enthalpy change of the hydride transfer reaction from one molar equivalent of hydride donor (XH) to one molar equivalent of hydride acceptor (Y^+^). Δ*H*_H_^−^_R_(XH) can be calculated by the following Equation (1), Δ*H*_H_^−^_R_(XH) = Δ*H*_H_^−^_A_(Y^+^) + Δ*H*_r_ (Equation (1)) [3,4]. Equation (1) allows us to determine the thermodynamic driving force for the release of hydrides from XH, which is a critical parameter for understanding the reducing abilities of XH and its potential applications in reduction reactions.

*Step 3* in Scheme 2 is the chemical process of XH releasing hydrogen atoms, XH → X^•^ + H^•^, and the thermodynamic driving force of *Step 2* is described as the molar enthalpy change of XH releasing hydrogen atoms, Δ*H*_HR_(XH), which is derived from Equation (2), Δ*H*_HR_(XH) = Δ*H*_H_^−^_R_(XH) − *F*[*E*_red_(X^+^) − *E*_red_(H^•^)] (Equation (2)), by constructing thermodynamic cycle I (*Step 3*–*Step 4*–*Step 2*) in Scheme 2 [3,29,30]. Among Equation (2), *E*_red_(H^•^) is reported as 1.128 V vs. Fc in acetonitrile and *F* is the faraday constant (23.06 kcal/V). It should be pointed out that the subscripted “HR” in Δ*H*_HR_ refers to hydrogen atoms releasing in the whole text.

*Step 5* in Scheme 2 is the chemical process of XH^•+^ releasing hydrogen atoms, described by the equation XH^•+^ → X^+^ + H^•^. The thermodynamic driving force of *Step 5* is the molar enthalpy change associated with the release of hydrogen atoms from XH^•+^, denoted as Δ*H*_HR_(XH^•+^). This value is derived from the following Equation (3), Δ*H*_HR_(XH^•+^) = Δ*H*_H_^−^_R_(XH) − *F*[*E*_ox_(XH) − *E*_red_(H^•^)] (Equation (3)), by constructing thermodynamic cycle II [3,29,30] (*Step 1*–*Step 5*–*Step 2*) in Scheme 2.

*Step 6* in Scheme 2 represents the chemical process of XH^•+^ releasing protons, described by the equation XH^•+^ → X^•^ + H^+^. The thermodynamic driving force of *Step 6* is described as the molar enthalpy change of XH^•+^ releasing protons, denoted as Δ*H*_PR_(XH^•+^). These values are derived from Equation (4), Δ*H*_PR_(XH^•+^) = Δ*H*_HR_(XH) − *F*[*E*_ox_(XH) − *E*_ox_(H^•^)] (Equation (4)). The equation is established by forming the thermodynamic cycle III [3,29,30] (*Step 1*–*Step 6*–*Step 3*) in Scheme 2.

*Step 7* in Scheme 2 is the chemical process of XH* releasing electrons, XH* → XH^•+^ + e. The thermodynamic driving force of *Step 7* is described as the oxidation potential of XH*, denoted as *E*_ox_(XH*). The oxidation potentials of the related photoexcited states BNAH*, HEH*, and AcrH* have been estimated as −2.98 V [31,32,33], −2.66 V [34], and −3.48 V [35,36] (V vs. Fc) respectively, which are available from published studies. In addition, the lifetimes of BNAH*, AcrH*, and HEH* are reported as 0.76 ns [33], 7 ns [5,35] and 0.32 ns [19], respectively. The *E*_ox_(XH*) values were typically obtained by integrating fluorescence or UV-vis with electrochemical technologies [32,37,38]. It should be noted that the potentials are converted from the standard calomel electrode (SCE) to ferrocene (Fc) by subtracting 0.380 V, based on the standard potentials of SCE and Fc in acetonitrile [39].

*Step 8* in Scheme 2 is the chemical process of XH* releasing hydrides or electron-coupled hydrogen atoms (ECH), XH* → X^+^ + H^−^ and XH* → X^+^ + ECH, and the thermodynamic driving force of *Step 8* is described as the molar enthalpy change associated with the release of hydrides or ECH from XH*, denoted as Δ*H*_H_^−^_R_(XH*). Given that the electron-donating ability of XH* is significantly enhanced upon photoexcitation, Δ*H*_H_^−^_R_(XH*) can be calculated from Δ*H*_H_^−^_R_(XH) and *E*_ox_(XH*) using Equation (5), Δ*H*_H_^−^_R_(XH*) = Δ*H*_H_^−^_R_(XH) − *F*[*E*_ox_(XH) − *E*_ox_(XH*)] (Equation (5)), according to Hess’ law.

*Step 9* in Scheme 2 is the chemical process of XH* releasing hydrogen atoms or proton-coupled electrons (PCE), XH* → X^•^ + H^•^ and XH* → X^•^ + PCE. The thermodynamic driving force of *Step 9* is described as the molar enthalpy change of XH* releasing hydrogen atoms or PCE, Δ*H*_HR_(XH*). These values were derived from Equation (6), Δ*H*_HR_(XH*) = Δ*H*_H_^−^_R_(XH*) − *F*[*E*_red_(X^+^) − *E*_red_(H^•^)] (Equation (6)), by constructing thermodynamic cycle IV [3,29,30] (*Step 9*–*Step 4*–*Step 8*) in Scheme 2.

Note that we used Gibbs free energy change ∆*G*_et_, ∆*G*_et_ = *F*∆*E*, to replace the enthalpy change ∆*H*_et_ in Equations (2)–(6) for the electron transfer processes. This substitution is suitable due to the fact that the entropy changes associated with electron transfer are negligible, as verified by Arnett et al. [29,30,40]. In our previous work, employing the same processing strategy, the thermodynamic driving forces for many organic hydrides releasing hydrogen atoms, and organic hydrides radical cations releasing hydrogen atoms or protons were derived by Equations (2)–(4) to characterize the reducing abilities of organic hydrides and related intermediates in acetonitrile [3].

### 2.2. Establishing Thermodynamic Cards of XH and XH* Releasing Hydrides in Acetonitrile

**Scheme 3:**
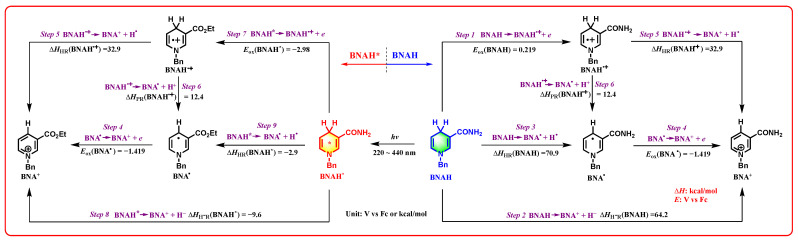
Thermodynamic card of BNAH and BNAH* releasing hydrides in acetonitrile.

**Scheme 4:**
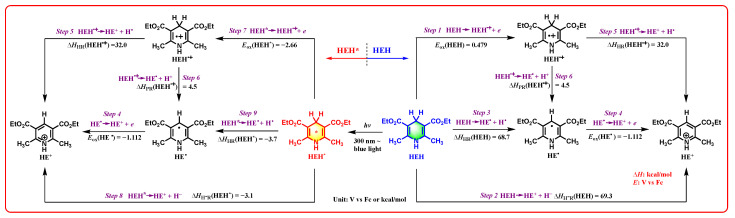
Thermodynamic card of HEH and HEH* releasing hydrides in acetonitrile.

**Scheme 5:**
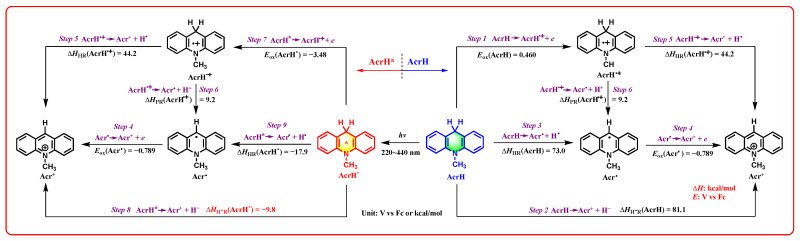
Thermodynamic card of AcrH and AcrH* releasing hydrides in acetonitrile.

Herein, the thermodynamic cards in nine potential elementary steps of BNAH, HEH, and AcrH and their related single excited states (BNAH*, HEH*, and AcrH*) releasing hydrides were established and shown in Scheme 3, Scheme 4 and Scheme 5, respectively. Additionally, all the relevant thermodynamic data have been compiled and presented in Table 1 for ease of reference.

### 2.3. Thermodynamic Abilities of XH* and XH Releasing Electrons

**Scheme 6:**
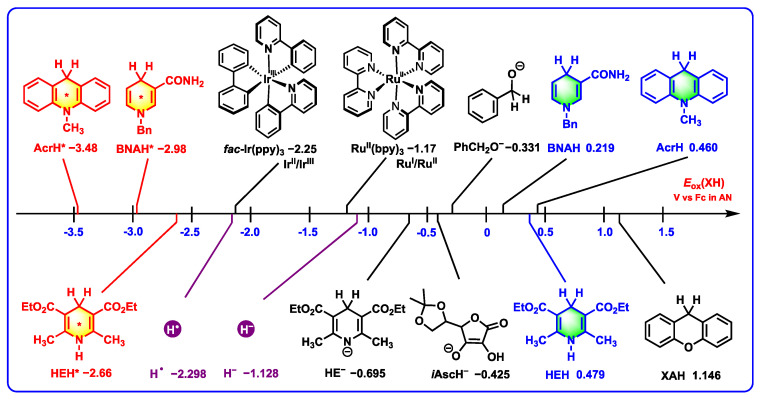
Comparisons of oxidation potentials of XH* and related XH, along with the oxidation potentials of common electron donors in acetonitrile (reference electrode Ag/AgNO_3_).

To facilitate a clear comparison of the oxidation potentials of XH and XH* with those of common single electron donors, the *E*_ox_(XH) and *E*_ox_(XH*) values (vs. Fc), as well as oxidation potentials of common organic hydrides and frequently-used photocatalyst metal complexes are shown in Scheme 6.

As illustrated in Scheme 6, the oxidation potential is 0.219 V for BNAH, 0.479 V for HEH, and 0.460 V for AcrH [3,4]. The thermodynamic electron-releasing abilities decrease in the order of BNAH (0.219 V) > AcrH (0.460 V) > HEH (0.479 V). Upon photoexcitation, the oxidation potentials for the excited states are significantly altered: −2.98 V for BNAH* [31,32,33], −2.66 V for HEH*^34^ and −3.48 V for AcrH* [35,36]. The order of their thermodynamic electron-donating abilities is substantially changed, with the sequence now being AcrH* (−3.48 V) > BNAH* (−2.98 V) > HEH* (−2.66 V). This indicates that photoexcitation dramatically enhances the electron-donating ability of these compounds, with AcrH* exhibiting the strongest thermodynamic propensity to release electrons among the three.

For the oxidation potentials of XH and XH*, it is observed that the *E*_ox_(XH) values of BNAH (0.219 V vs. Fc), HEH (0.479 V vs. Fc), and AcrH (0.460 V vs. Fc) are all greater than 0 V and less than 0.5 V (vs. Fc), 0 < *E*_ox_(XH) < 0.5 V. In contrast, the *E*_ox_(XH*) values of BNAH* (−2.98 V vs. Fc), HEH* (−2.66 V vs. Fc) and AcrH* (−3.48 V vs. Fc) are significantly more negative than −1.0 V (vs. Fc) and even more negative than that of *E*_ox_(H^•^) (−2.298 V vs. Fc in acetonitrile). This indicates that BNAH, HEH, and AcrH are classified as thermodynamically medium-strong single electron donors [3], whereas their photoexcited counterparts (BNAH*, HEH* and AcrH*) are thermodynamically very strong single electron donors.

When comparing the photoexcited oxidation potentials of BNAH* (−2.98 V vs. Fc), HEH* (−2.66 V vs. Fc) and AcrH* (−3.48 V vs. Fc) with their parents (BNAH, HEH, and AcrH), it is found that the oxidation potential decreases by 3.199 V for BNAH*, 3.139 V for HEH*, and 3.940 V for AcrH*. This suggests that the photoexcitation greatly improves the electron-releasing abilities by at least 72.4 kcal/mol (72.4 kcal/mol for HEH*, 73.8 kcal/mol for BNAH* and 90.9 kcal/mol for AcrH*).

As electron donors, the thermodynamic abilities of XH* and XH to release electrons decrease in the following order of AcrH* (−3.48 V vs. Fc) > BNAH* (−2.98 V vs. Fc) > HEH* (−2.66 V vs. Fc) > BNAH (0.219 V vs. Fc) > AcrH (0.460 V vs. Fc) > HEH (0.479 V vs. Fc). Notably, although the oxidation potentials of HEH (0.479 V vs. Fc) and AcrH (0.460 V vs. Fc) are very close, the electron-releasing ability of AcrH* (80.2 kcal/mol refer to Fc) is 18.9 kcal/mol greater than that of HEH* (61.3 kcal/mol refer to Fc) after photoexcitation.

It is well established that *fac*-Ir(ppy)_3_ and Ru^II^(bpy)_3_ are extensively employed in photocatalytic reactions [41,42,43,44,45], where their key intermediates (Ir^III^ and Ru^I^) serve as highly effective electron donors to activate reaction substrates. If the *E*_ox_(XH) and *E*_ox_(XH*) values are compared with *E*_ox_[Ir^II^(ppy)_3_] (−2.25 V vs. Fc) and *E*_ox_[Ru^I^(bpy)_3_] (−1.17 V vs. Fc) [46], it is evident that *E*_ox_(XH) values (0.219 – 0.479 V vs. Fc) are significantly more positive than that of *E*_ox_[Ru^I^(bpy)_3_] (−1.17 V vs. Fc). Conversely, the *E*_ox_(XH*) values (−2.66 – −3.48 V vs. Fc) are much more negative than that of *E*_ox_[Ir^II^(ppy)_3_] (−2.25 V vs. Fc). This comparison indicates that upon photoexcitation, XH* exhibit even stronger thermodynamic single-electron donating abilities than common photocatalysts. Therefore, XH* can serve as superior alternatives to metal-complex photocatalysts [11,12,13,14,15,16], offering the advantage of extremely low oxidation potentials and eliminating the need of additional electron-donating additives.

### 2.4. Thermodynamic Abilities of XH* and XH Releasing Hydrides

**Scheme 7:**
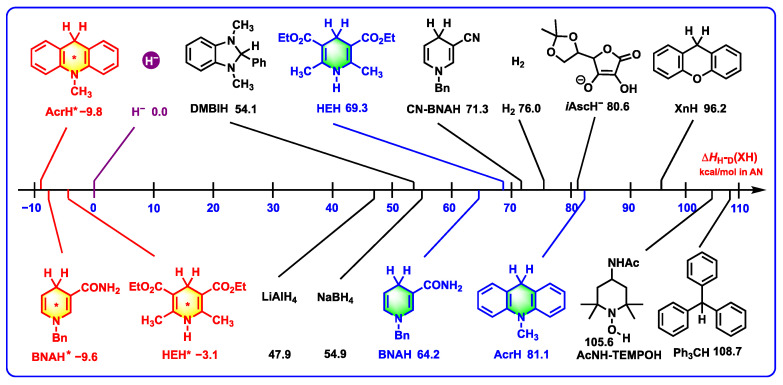
Comparisons of hydride-releasing abilities of XH* and related XH, along with the hydride-releasing abilities of common hydride donors in acetonitrile.

For a more comprehensive comparison, the thermodynamic driving forces associated with the release of hydrides from XH* and related XH, as well as those from common hydride donors, are exhibited in Scheme 7. It is well known that the NADH coenzyme acts as a medium-strong hydride carrier in biological systems, playing an essential role in mediating the transfer of hydrides between substrates and oxidoreductases [1,2,3,4]. Given its structural similarity to NADH, BNAH (64.2 kcal/mol) is considered a thermodynamically medium-strong hydride donor. Similarly, HEH (69.3 kcal/mol) also falls into the category of thermodynamically medium-strong hydride donors [4].

In contrast, AcrH has a significantly weaker thermodynamic ability to release hydrides, being 16.9 kcal/mol less favorable than BNAH (64.2 kcal/mol). As a result, AcrH is recognized as a thermodynamically weak hydride donor. In summary, BNAH (64.2 kcal/mol), HEH (69.3 kcal/mol), and AcrH (81.1 kcal/mol) belong to thermodynamically medium-strong or weak hydride donors individually [3], and the thermodynamic abilities of XH releasing hydrides decrease in the order of BNAH (64.2 kcal/mol, medium-strong) > HEH (69.3 kcal/mol, medium-strong) > AcrH (81.1 kcal/mol, weak).

From Scheme 7, it is clear that the thermodynamic driving force of XH* releasing hydrides is −9.6 kcal/mol for BNAH*, −3.1 kcal/mol for HEH*, −9.8 kcal/mol for AcrH* respectively. The thermodynamic hydride-donating abilities decrease in the following order of AcrH* (−9.8 kcal/mol) ≈ BNAH* (−9.6 kcal/mol) > HEH* (−3.1 kcal/mol). In the published literature, LiAlH_4_ is reported as a very strong inorganic hydride donor, with a thermodynamic driving force of 47.9 kcal/mol for hydride release [47]. In contrast, the thermodynamic driving forces of XH* releasing hydrides range from −3.1 kcal/mol to −9.8 kcal/mol, which are significantly more negative than that of LiAlH_4_ (47.9 kcal/mol) [47]. This indicates that XH* are exceptionally strong hydride donors from a thermodynamic standpoint.

Moreover, if the hydride-donating abilities of XH* are compared with free hydride ions (H^−^, 0.0 kcal/mol), it is found that AcrH* (−9.8 kcal/mol), BNAH* (−9.6 kcal/mol), and HEH* (−3.1 kcal/mol) are even thermodynamically more favorable hydride donors than free hydride ions (H^−^, 0.0 kcal/mol) in acetonitrile. This remarkable thermodynamic advantage suggests that XH* could serve as highly efficient hydride carriers to activate or reduce challenging substrates in chemical reactions. If the thermodynamic driving forces of XH* (−3.1–−9.8 kcal/mol) releasing hydrides are compared with those of their ground-state parents XH (64.2–81.1 kcal/mol), it is evident that the hydride-donating abilities of XH* are greatly improved by the photoexcitation. As hydride donors, the thermodynamic abilities of XH* and XH releasing hydrides decrease in the order of AcrH* (−9.8 kcal/mol) ≈ BNAH* (−9.6 kcal/mol) > HEH* (−3.1 kcal/mol) > BNAH (64.2 kcal/mol) > HEH (69.3 kcal/mol) > AcrH (81.1 kcal/mol).

As indicated in Scheme 3, Scheme 4 and Scheme 5, an intriguing observation emerges regarding the thermodynamic driving forces associated with the release of electrons from XH*. Specifically, the thermodynamic driving forces of XH* (−2.66 – −3.48 V vs Fc) releasing electrons are greater than −61.3 kcal/mol (referring to Fc, −2.66 V vs. Fc), while the following thermodynamic driving forces of XH^•+^ (32.0 – 44.2 kcal/mol) releasing hydrogen atoms are smaller than 32.0 kcal/mol. These thermodynamic data strongly suggest that the rate of electron release is likely diffusion-controlled and several orders of magnitude faster than the subsequent hydrogen transfer rate. This disparity in rates clearly indicates that the overall hydride transfer process may proceed via a concerted electron-coupled hydrogen atom (ECH) transfer mechanism in a single step [27]. The thermodynamic analysis of this process mirrors that of XH* releasing hydrides directly, highlighting the significant role of electron coupling in facilitating the hydrogen atom transfer.

### 2.5. Thermodynamic Abilities of XH* and XH Releasing Hydrogen Atoms

**Scheme 8:**
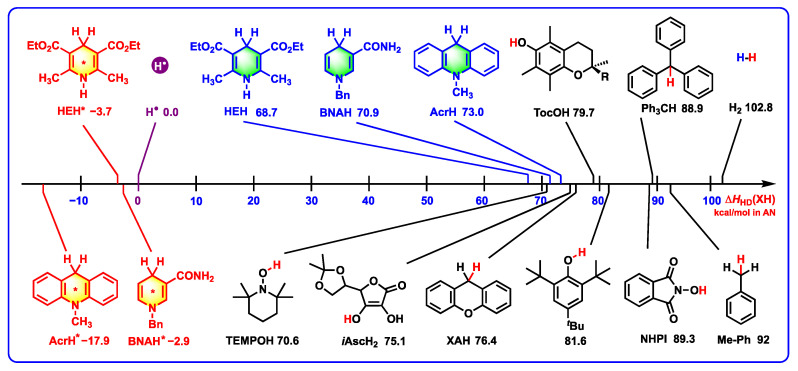
Comparisons of hydrogen atom-releasing abilities of XH* and related XH, along with that of common hydrogen atom donors in acetonitrile (R group in TocOH refers to (4*R*,8*R*)-4,8,12-trimethyltridecyl).

The thermodynamic hydrogen atom-releasing abilities of XH* and related XH, as well as those of common hydrogen atom donors, are presented in Scheme 8. Since ascorbic acid (AscH_2_) and α-tocopherol (TocOH) are reported as well-known antioxidants in biological systems, the molar enthalpy changes of 5,6-isopropylidene ascorbic acid (*i*AscH_2_) and TocOH releasing hydrogen atoms are determined as 75.1 kcal/mol and 79.7 kcal/mol in previous studies in acetonitrile [48], which are shown in Scheme 8 for comparison.

From Scheme 8, it is evident that the thermodynamic driving forces of BNAH (70.9 kcal/mol), HEH (68.7 kcal/mol), and AcrH (73.0 kcal/mol) releasing hydrogen atoms are smaller than that of *i*AscH_2_ (75.1 kcal/mol) and TocOH (79.7 kcal/mol) [48]. This implies that BNAH (70.9 kcal/mol), HEH (68.7 kcal/mol), and AcrH (73.0 kcal/mol) are thermodynamically more favorable antioxidants than *i*AscH_2_ (75.1 kcal/mol) [48] and TocOH (79.7 kcal/mol) [48] in acetonitrile. The thermodynamic abilities of XH releasing hydrogen atoms decrease in the following order of HEH (68.7 kcal/mol) > BNAH (70.9 kcal/mol) > AcrH (73.0 kcal/mol).

Additionally, upon photoexcitation, the thermodynamic driving forces of HEH* (−3.7 kcal/mol), BNAH* (−2.9 kcal/mol), and AcrH* (−17.9 kcal/mol) releasing hydrogen atoms are significantly more negative than that of free hydrogen atoms (H^•^, 0.0 kcal/mol) in acetonitrile. This illustrates that HEH* (−3.7 kcal/mol), BNAH* (−2.9 kcal/mol), and AcrH* (−17.9 kcal/mol) are exceptionally strong hydrogen atom donors or antioxidants from a thermodynamic perspective. The thermodynamic abilities of XH* to release hydrogen atoms decrease in the following order: AcrH* (−17.9 kcal/mol) > HEH* (−3.7 kcal/mol) > BNAH* (−2.9 kcal/mol).

Ultimately, if the thermodynamic driving forces of XH* and XH releasing hydrogen atoms are compared, it is found that the thermodynamically hydrogen atom-donating abilities of XH* are greatly improved by photoexcitation. As hydrogen atom donors, the thermodynamic abilities of XH* and XH releasing hydrogen atom decrease in the order of AcrH* (−17.9 kcal/mol) > HEH* (−3.7 kcal/mol) > BNAH* (−2.9 kcal/mol) > HEH (68.7 kcal/mol) > BNAH (70.9 kcal/mol) > AcrH (73.0 kcal/mol).

Since XH* are much more effective hydrogen atom donors than their parents XH, the thermodynamic data lead us to take full advantage of the thermodynamic abilities of XH* in chemical reactions. For certain challenging unsaturated substrates (S), the direct hydrogen atoms transfer from XH to S is thermodynamically unfeasible, as represented by the reaction XH + S → X^•^ + SH^•^. In such cases, chemists could seek the help of photoexcitation to convert XH into its excited XH* thereby facilitating the hydrogen atom transfer to the substrate, XH* + S → X^•^ + SH^•^ [8]. This strategy has been previously demonstrated to be effective. The thermodynamic driving forces associated with these processes provide critical insights. Specifically, the oxidation potentials of XH* are less than −2.66 V (vs. Fc), which corresponds to a thermodynamic driving force of −61.3 kcal/mol. In contrast, the subsequent thermodynamic driving forces for the release of protons from XH^•+^ range from 4.5 kcal/mol to 12.4 kcal/mol. These thermodynamic data suggest that the electron-releasing rate may be diffusion-controlled and several orders of magnitude larger than the following protons transfer rate, which clearly demonstrates that the overall hydrogen atom transfer process may undergo proton-coupled electron transfer (PCET) to activate unsaturated substrates [8,49,50,51] and the corresponding thermodynamic ability analysis of PCET is the same to that of XH* releasing hydrogen atoms.

### 2.6. Application of Thermodynamic Cards of XH and XH* to Reduction Reactions

**Scheme 9:**
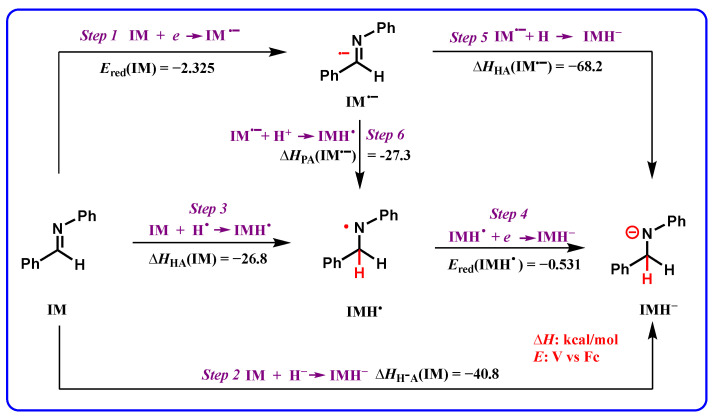
Thermodynamic card of N,1-diphenylmethanimine (IM) accepting hydrides in acetonitrile.

As discussed earlier, photoexcited XH* are considered thermodynamically better hydride carriers than their parents XH in chemical reactions [5,6,7,11,12,13,14,15,16,17,18,19,20,21,22,23,24,25,26]. To illustrate the application of the thermodynamic cards of XH and XH* in reduction reactions, the reductions of N,1-diphenylmethanimine (IM) by AcrH and AcrH* were examined. In our earlier work, the six thermodynamic driving forces of common imines accepting hydrides were determined in acetonitrile [52], and the thermodynamic card of N,1-diphenylmethanimine (IM) accepting hydrides is shown Scheme 9.

**Scheme 10:**
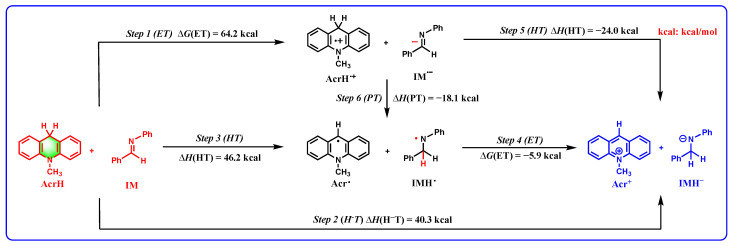
Thermodynamic analysis platform (TAP) of hydride transfer from AcrH to N,1-diphenylmethanimine (IM) in acetonitrile.

**Scheme 11:**
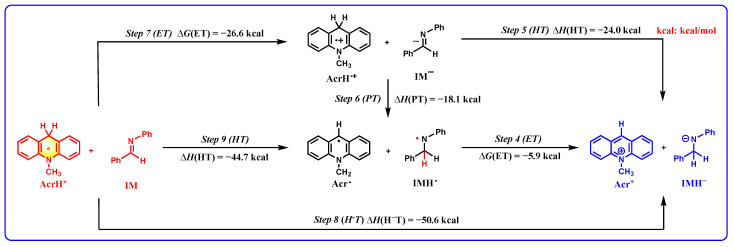
Thermodynamic analysis platform (TAP) of hydride transfer from AcrH* to N,1-diphenylmethanimine (IM) in acetonitrile.

Scheme 5 and Scheme 9 provide a comprehensive understanding of the thermodynamic feasibility of hydride transfer from various donors to IM, highlighting the potential advantages of using photoexcited hydride donors (XH*) over their ground-state counterparts (XH) in facilitating such reductions. Based on the thermodynamic cards of AcrH and AcrH* (Scheme 5), as well as IM (Scheme 9), the thermodynamic analysis platforms (TAP) [3,52] of hydride transfer from AcrH and AcrH* to N,1-diphenylmethanimine (IM) are shown in Scheme 10 and Scheme 11, respectively.

From Scheme 10, the hydride transfer from AcrH to IM may involve six possible elementary steps and experience four potential mechanisms, including e + H^•^ (*Step 1 + Step 5*), e + H^+^ + e (*Step 1 + Step 6 + Step 4*), H^•^ + e (*Step 3 + Step 4*), and H^−^ (*Step 2*) pathways. Considering the thermodynamic driving forces of six related elementary steps (*Step 1–Step 6* in Scheme 10), three potential initiating steps are identified, that is, electron transfer (*Step 1*), hydrogen atom transfer (*Step 3*), and hydride transfer (*Step 2*). It is found from Scheme 10 that the thermodynamic driving force is 64.2 kcal/mol for *Step 1* (ET), 46.2 kcal/mol for *Step 3* (HT), and 40.3 kcal/mol for *Step 2* (H^−^T). The thermodynamic driving forces for all three initiating steps (*Step 1–Step 3*) are greater than 40.0 kcal/mol, which are very high energy barriers to overcome. This indicates that the hydride transfer reaction from AcrH to IM is thermodynamically highly unfavorable or effectively impossible under these conditions.

In contrast, as can be seen from Scheme 11, the thermodynamic driving force for hydride transfer from AcrH* to IM is calculated to be −50.6 kcal/mol, based on the Δ*H*_H_^−^_R_(AcrH*) and Δ*H*_H_^−^_A_(IM) values. This negative value indicates the hydride transfer reaction from AcrH* to IM is thermodynamically feasible. Further analysis of the thermodynamic driving forces for the six possible elementary steps (*Step 4–Step 9* in Scheme 11) reveals that these values range from −5.9 kcal/mol to −50.6 kcal/mol, all of which are significantly negative. This demonstrates that each of these elementary steps is thermodynamically feasible. Moreover, the three possible elementary steps initiated, namely *Step 7* Δ*G*(ET) = −26.6 kcal/mol, *Step 8* Δ*H*(HT) = −44.7 kcal/mol, and *Step 9* Δ*H*(H^−^T) = −50.6 kcal/mol, have very large thermodynamic driving forces (<< −20 kcal/mol). Therefore, the following four mechanisms are all thermodynamically feasible for hydride transfer from AcrH* to IM: e + H^•^ (*Step 7 + Step 5*), e + H^+^ + e (*Step 7 + Step 6 + Step 4*), H^•^ + e (*Step 9 + Step 4*), and H^−^ (*Step 8*) mechanisms. In practice, BNAH*, AcrH* and HEH* have been widely applied into photochemical synthesis, and the process of XH* releasing hydrides in chemical reactions may involve multiple mechanisms (Scheme 1b), including e [11,12,13,14,15,16], e + H^•^ [17,18,19,20,21,22,23], e + H^+^ + e [24,25], H^•^ + e [26], H [9,10,27,28] mechanisms. As for which one is the true hydride reduction mechanism between IM and AcrH*, it needs further validation and support in the experiment.

In fact, AcrH itself (81.1 kcal/mol) is a weak organic hydride and is unable to reduce IM via hydride transfer [3]. Meanwhile, after photoexcitation, AcrH* becomes a very strong hydride reductant (−9.8 kcal/mol), capable of easily reducing IM through hydride transfer. This highlights the significant impact of photoexcitation on enhancing the hydride-donating ability of XH*, transforming the hydride transfer reaction from thermodynamically unfeasible to feasible.

## 3. Experimental Section

### 3.1. Electrochemical Experiments

The potential determinations were carried out by CV and OSWV methods simultaneously via an electrochemical apparatus (BAS-100B) under the protection of Ar or N_2_ in the deaerated acetonitrile. The standard three-electrode system was used as follows: working electrode (glassy carbon), and reference electrode (0.01 M Ag/AgNO_3_ in 0.1 M ^n^Bu_4_NPF_6_/AN solution), as well as auxiliary electrode (platinum wire). The Fc/Fc^+^ couple was used as a reference for all measurements. Scans rate is 100 mV s^−1^. The concentration of the XH or X^+^ was ~1 mM in 0.1 M nBu_4_NPF_6_/AN solution. The estimated determination errors were smaller than 5 mV. Detailed electrochemical experiments, as well as the *E*_ox_(XH) values of *Step 1* and *E*_red_(X^+^) values of *Step 4* for BNAH, HEH and AcrH in acetonitrile can be found in our previous work [3,4].

### 3.2. Isothermal Titration Experiments

The Δ*H*_H_^−^_R_(XH) values of *Step 2* in acetonitrile were determined in our previous work by isothermal titration experiments [3,4]. The isothermal titration experiments were performed through a CSC4200 isothermal titration calorimeter (ITC) at 298 K in acetonitrile. The corresponding XH and Y^+^ were dissolved in the deaerated acetonitrile with a certain concentration, *C*_XH_ and *C*_Y_^+^. An injection of XH (10.0 μL) into Y^+^ solution was delivered in 1 s, and the time interval between every two injections was 300~500 s. Injections (denoted *n* times) were repeated over 10 times for each isothermal titration spectrum, and the heat changes were collected by an ITC detector. The molar reaction heat (Δ*H*_r_) between XH and Y^+^ was calculated by integrating every peak except the first one of the isothermal titration spectrum (denoted as Δ*H_int_*) by combing the known reaction concentration of XH (*C*_XH_) and injection times (*n*) through equation Δ*H*_r_ = Δ*H_int_*/(*n* × 10 uL × *C*_XH_).

### 3.3. The E_ox_(XH*) Values Determination

The *E*_ox_(XH*) values are obtained by a combination of fluorescence and electrochemical technologies for BNAH* [31,32,33] and AcrH* [35,36]. The maximum emission wavelengths of XH*, λ_max_(em), were determined by a fluorescence spectrophotometer, which are the wavelengths of the strongest or highest peak from the related fluorescence emission spectrum. The excited-state energy of XH*, *E*_00_(XH*) (*ev*), was derived from λ_max_(em) of the XH* luminescence spectrum. The *E*_ox_(XH*) was obtained by subtracting the *E*_00_(XH*) from *E*_ox_(XH), *E*_ox_(XH*) = *E*_ox_(XH) − *E*_00_(XH*) [32]. In fact, *E*_00_ of photoexcited compounds can be estimated in a few different ways [41,42,43]. Similarly, the *E*_ox_(HEH*) value was derived from a combination of the UV-vis absorption spectrum of HEH and *E*_ox_(HEH) [34,41,42,43]. The *E*_ox_(XH*) values of *Step 7* for BNAH* (−2.98 V vs. Fc) [31,32,33], HEH* (−2.66 V vs. Fc) [34,41,42,43] and AcrH* (−3.48 V vs. Fc) [35,36] are available in published work.

## 4. Conclusions

In summary, this work constructs detailed thermodynamic cards for three classic NADH models (BNAH, HEH, and AcrH), as well as their photoexcited states (BNAH*, HEH*, and AcrH*) in acetonitrile, focusing on their ability to release hydrides. The analysis reveals that photoexcitation significantly enhances the reducing abilities of these compounds, making XH* exceptionally strong electron, hydrogen atom, and hydride donors compared to their ground-state counterparts (XH).

Specifically, as electron donors, the thermodynamic abilities of XH* and XH to release electrons decrease in the order of AcrH* (−3.48 V vs. Fc) > BNAH* (−2.98 V vs. Fc) > HEH* (−2.66 V vs. Fc) > BNAH (0.219 V vs. Fc) > AcrH (0.460 V vs. Fc) > HEH (0.479 V vs. Fc). As hydride donors, the thermodynamic abilities of XH* and XH to release hydrides decrease in the order of AcrH* (−9.8 kcal/mol) ≈ BNAH* (−9.6 kcal/mol) > HEH* (−3.1 kcal/mol) > BNAH (64.2 kcal/mol) > HEH (69.3 kcal/mol) > AcrH (81.1 kcal/mol). As hydrogen atom donors, the thermodynamic abilities of XH* and XH to release hydrogen atom decrease in the order of AcrH* (−17.9 kcal/mol) > HEH* (−3.7 kcal/mol) > BNAH* (−2.9 kcal/mol) > HEH (68.7 kcal/mol) > BNAH (70.9 kcal/mol) > AcrH (73.0 kcal/mol). Among the three classic NADH models, AcrH* emerges as the thermodynamically superior donor of electrons, hydrides, and hydrogen atoms. Its exceptional reducing abilities highlight the potential of photoexcited organic hydrides in facilitating challenging reductions and activating inert substrates.

These findings suggest that NADH models, particularly their photoexcited states, may have broader applications in organic synthesis and photochemical reactions due to their enhanced reducing capabilities. The impressive thermodynamic cards of XH* inspire further investigation into the excited wavelengths, excited potentials, and excited-state stabilities of more organic hydrides. This research direction holds promise for discovering novel and highly effective photoexcited organic hydride reductants with potential applications in various chemical transformations.

## Figures and Tables

**Table 1:** Thermodynamic data of nine potential elementary steps of BNAH, HEH, and AcrH and their related single excited states (BNAH*, HEH*, and AcrH*) releasing hydrides in acetonitrile.

Step X	Process	Parameters	Unit	BNAH	HEH	**AcrH**
*Step 1*	XH → XH^•+^ + e	*E*_ox_(XH)	V vs. Fc	0.219	0.479	0.460
*Step 2*	XH → X^+^ + H^−^	Δ*H*_H_^−^_R_(XH)	kcal/mol	64.2	69.3	81.1
*Step 3*	XH → X^•^ + H^•^	Δ*H*_HR_(XH)	kcal/mol	70.9	68.7	73.0
*Step 4*	X^•^ → X^+^ + e	*E*_ox_(X^•^)	V vs. Fc	−1.419	−1.112	−0.789
*Step 5*	XH^•+^ → X^+^ + H^•^	Δ*H*_HR_(XH^•+^)	kcal/mol	32.9	32.0	44.2
*Step 6*	XH^•+^ → X^•^ + H^+^	Δ*H*_PR_(XH^•+^)	kcal/mol	12.4	4.5	9.2
*Step 7*	XH* → XH^•+^ + e	*E*_ox_(XH*)	V vs. Fc	−2.98	−2.66	−3.48
*Step 8*	XH* → X^+^ + H^−^ XH* → X^+^ + ECH	Δ*H*_H_^−^_R_(XH*)	kcal/mol	−9.6	−3.1	−9.8
*Step 9*	XH* → X^•^ + H^•^ XH* → X^•^ + PCE	Δ*H*_HR_(XH*)	kcal/mol	−2.9	−3.7	−17.9

## Data Availability

Data are contained within the article.
